# Interchange of Southern Hemisphere humpback whales across the South Atlantic Ocean

**DOI:** 10.1038/s41598-023-31358-5

**Published:** 2023-03-21

**Authors:** Eric Angel Ramos, Ted Cheeseman, Milton Cesar C. Marcondes, Marilia Olio, Alexander Vogel, Simon Elwen, Thais H. M. de Melo, Cecília Facchola, Sérgio Cipolotti, Ken Southerland, Ken Findlay, Elisa Seyboth, Steven A. McCue, Pieter G. H. Kotze, S. Mduduzi Seakamela

**Affiliations:** 1 Fundación Internacional para la Naturaleza y la Sustentabilidad, Chetumal, Quintana Roo Mexico; 2 Happywhale, Santa Cruz, CA USA; 3 Marine Ecology Research Centre, Southern Cross University, Lismore, Australia; 4 Instituto Baleia Jubarte, Caravelas, Bahia Brazil; 5 Seafari App, Cape Town, South Africa; 6 Department of Botany and Zoology, Stellenbosch University, Sea Search Research and Conservation, Cape Town, South Africa; 7 Cia do Mar & Baleia à Vista, Porto Seguro, Bahia Brazil; 8 Faculty of Applied Sciences, Centre for Sustainable Oceans, Cape Peninsula University of Technology, Cape Town, South Africa; 9 Department of Forestry, Fisheries and the Environment, Branch Oceans and Coasts, V& A Waterfront, Cape Town, South Africa

**Keywords:** Ocean sciences, Zoology, Animal behaviour

## Abstract

The cosmopolitan distribution of humpback whales (*Megaptera novaeangliae*) is largely driven by migrations between winter low-latitude breeding grounds and summer high-latitude feeding grounds. Southern Hemisphere humpback whales faced intensive exploitation during the whaling eras and recently show evidence of population recovery. Gene flow and shared song indicate overlap between the western (A) and eastern (B1, B2) Breeding Stocks in the South Atlantic and Indian Oceans (C1). Here, we investigated photo-identification evidence of population interchange using images of individuals photographed during boat-based tourism and research in Brazil and South Africa from 1989 to 2022. Fluke images were uploaded to Happywhale, a global digital database for marine mammal identification. Six whales were recaptured between countries from 2002 to 2021 with resighting intervals ranging from 0.76 to 12.92 years. Four whales originally photographed off Abrolhos Bank, Brazil were photographed off the Western Cape, South Africa (feeding grounds for B2). Two whales originally photographed off the Western Cape were photographed off Brazil, one traveling to the Eastern Cape in the Southwestern Indian Ocean (a migration corridor for C1) before migrating westward to Brazil. These findings photographically confirm interchange of humpback whales across the South Atlantic and Indian Oceans and the importance of international collaboration to understand population boundaries.

## Introduction

The conservation and management of migratory species that traverse ocean basins is complex and depends on an understanding of population boundaries and interconnectivity to accurately identify areas of importance for their breeding and feeding activities^1^. Additionally, as animals cross national and international boundaries and areas beyond national jurisdiction, collaboration between a wide array of stakeholders is essential to global understanding^2^. This information is particularly important for populations recovering from major historical impacts, such as the extensively hunted large baleen whales^3,4^.

Humpback whales (*Megaptera novaeangliae*) are globally distributed in all oceans, with most populations displaying stable seasonal migratory routes between high-latitude feeding grounds and low-latitude breeding grounds^5,6^. At an ocean-basin scale, the genetic structure of humpback whale populations is a result of fidelity to specific feeding grounds and natal philopatry to breeding grounds^7,8^. Extensive data from whaling eras and whaling related research (e.g., the Discovery Marking Programme^9^), coupled with global genetic studies and photographic capture-recapture of whales, broadly support the traditionally recognized populations or ‘stocks’ of humpback whales, as they are termed by the International Whaling Commission (IWC), and their distinct distribution patterns^6,10^. However, increasingly studies using both photo-identification (photo-ID)^10,11^, satellite telemetry^12^, and evidence for cultural exchange of humpback whale song (typically shared within populations and spread regionally)^13,14^ also support that population boundaries can be porous, and that exchange does occur between recognized stocks.

Photo-ID of humpback whales since the 1970’s has provided a powerful tool for investigating population demography and migratory movements of whales in different regions^15,16^. The development of global networks of research collaborations and citizen science data collection of humpback whale fluke photos, as well as advancements in automated imaging techniques^17^ have led to the discovery of numerous instances of humpback whales documented migrating outside of their expected ranges. These records include the detection of whales thousands of kilometers from their expected migratory location^18–20^, and include transoceanic movements between multiple breeding grounds^19,21^ and between breeding and feeding grounds^22–26^; the use of multiple distinct breeding grounds in the same season^26,27^; migrations to areas beyond the known range of their stock^28^; and feeding in known breeding grounds^29^.

In the Southern Hemisphere, humpback whales were the target of intensive commercial whaling in the early twentieth century at both their breeding and feeding grounds, as well as along their migratory routes. Along the Atlantic coast of Brazil, pre-modern whaling was at its peak in the eighteenth century, leading to a drastic reduction of whale populations near the Antarctic and South Georgia islands to several hundred whales^3,30^. The hunting of humpback whales was prohibited in 1966 by the IWC in the Southern Ocean, but illegal Soviet whaling persisted until the moratorium on commercial whaling was put in place in 1986^3,4,31^. The cessation of whaling for over four decades has allowed global recovery of many humpback whale populations^30,32^. There have been noted increases in the abundance of Southern Hemisphere humpback whales in the Western South Atlantic Ocean^33–36^ and Southwestern Indian Ocean^33,35–37^ with population increases in the region of 7–10% per annum.

The IWC recognizes seven Breeding Stocks of humpback whales (A through G) found off the continental coasts in the Southern Ocean^38^. Each stock is also affiliated with areas a high latitude feeding grounds in the Southern Ocean, designated as Areas I through VI and likely migratory corridors that connect these^31^. In the South Atlantic Ocean, humpback whales are divided into two distinct breeding populations found on opposite sides of the ocean basin. Whales of stock A winter off the coast of Brazil in South America^39,40^ and migrate south to summer feeding grounds off South Georgia^41^ and the Antarctica Peninsula^32,42–44^. On the African coast, whales of stock B are divided into substocks B1, with a breeding ground in the Gulf of Guinea and feeding grounds in Antarctic Area III. Substock B2 uses the west coast of southern Africa as a feeding ground^45^. However, the breeding grounds^46–48^ and overall spatial and temporal distribution of substock B2 remains poorly understood^49^. Whales from stock C are recognized to feed in Antarctic Area III as well. Four different substocks breed on the east coast of Africa and western Indian Ocean, divided into substocks C1 off Mozambique, C2 in the central Mozambique Channel Islands, C3 along the southeast coast of Madagascar, and C4 around the Mascarene Islands^47,50^.

In recent years, different lines of evidence have indicated connectivity between these recognized stocks. This connectivity between whale populations in the eastern and western South Atlantic Ocean and Southwestern Indian Ocean was initially established from analysis of whale song^12,13^. The intermixing of whales of stocks A and B has also been indicated from evidence of low levels of gene flow, and the occurrence of the same song between populations in Gabon and Brazil^13,24^. Similarly, a genetic recapture of a whale that was sampled in both Gabon and Madagascar revealed its travel between breeding areas associated with substocks B1 and C3^23^ while a photographic recapture linked stock B1 in Brazil to substock C3 in Madagascar^26^. Ross-Marsh et al.^51^ and Schall et al.^14^ recently showed regular and ongoing exchange of culturally learned song units by humpback whales across breeding, migratory, and feeding areas, likely driven mainly by overlap in the Southern Ocean feeding grounds but potentially driven by relatively few individuals. Their findings and genetic analysis of Southern Ocean humpback whale populations further support population mixing over multiple years at feeding grounds in the Atlantic sector of the Southern Ocean (ASSO) off Antarctica^52^. Additionally, a global data set of satellite telemetry data shows a high degree of overlap of tagged animals from stocks A, B1, B2, and C in the ASSO, although most of these movements are limited to ‘one-way’ movements across a single season^53^.

An improved understanding of how often transoceanic migration events occur in humpback whale populations, and the ways in which these interpopulation exchanges are associated with climate change related impacts and/or their recovery from commercial whaling, demands better long-term tracking of individual whale movements across populations. Here, we report the photographic recapture of six Southern Hemisphere humpback whales across the South Atlantic Ocean from Brazil to the west coast of South Africa, one of which was also photographed in the Indian Ocean on the east coast of South Africa.

## Materials and methods

### Data collection

Data for this study included photographs collected during humpback whale sightings off the Abrolhos Bank of Brazil in the Southwestern Atlantic Ocean and the west (Southeastern Atlantic Ocean) and east (Southwestern Indian Ocean) coasts of South Africa. In all cases, photos of the ventral side of humpback whale flukes were taken with digital SLR cameras equipped with telephoto lenses.

In Brazil, data were collected during dedicated boat-based surveys and commercial whale watch cruises (90–180 min long) conducted throughout the breeding season from 1989 to 2022. Surveys operated along a stretch of ~ 2200 km off the Atlantic coast of Brazil in the states of Bahia, São Paulo, Espírito Santo, Rio de Janeiro, Rio Grande do Sul, Paraná, and Santa Catarina by Instituto Baleia Jubarte (IBJ) (3 to 4 cruises per week) and the commercial whale-watching company Cia do Mar & Baleia à Vista (30 to 60 cruises per year) from early July to the end of October between 2008 and 2021 (excluding 2013 and 2020).

Data from South Africa were provided by scientific research groups, commercial whale-watching operators, and citizens during boat-based and land-based efforts from 2002 to 2022. Whale data were also gathered during dedicated research surveys by the Department of Forestry, Fisheries and the Environment (DFFE) of the government of South Africa, Sea Search Research and Conservation of the University of Stellenbosch, and Cape Peninsula University of Technology (CPUT) through activities of the Whales and Climate Research Project. Many sightings, including some from citizen science, were uploaded through the Seafari App (www.seafariapp.org), a mobile phone application enabling users to log marine mammal sightings^54^. Fluke images were subsequently collated and verified by author AV and uploaded to Happywhale.

Humpback whale fluke photographs and metadata associated with sightings gathered during boat- and land-based observations concurrent with photo-ID efforts and images were uploaded to the Happywhale platform (www.happywhale.com,^17^). All data were compiled to characterize data collection in both regions and to identify potential recaptures of individually identified humpback whales across the South Atlantic Ocean and the Southwest Indian Ocean. The images we used had been previously categorized by quality and the highest quality image per individual was used to construct a unique photo-ID catalog for each collaborating institution or individual.

### Data analysis

All selected fluke images and supporting sighting data were aggregated into a standardized database in the Happywhale platform. Fluke images were comprehensively matched within a global database of the flukes of 69,997 individually distinct humpback whales as of 05 Aug 2022 (as its a growing catalog). Matching was undertaken via automated image recognition, with each potential match further verified by a trained observer. There was an expected accuracy (false positives and false negatives) of 97–99% of potential matches found after human verification^55^.

Sighting data from Happywhale were processed in *R* statistical software^56^ to determine the number of sightings of individually identified humpback whales per year in each location. The results were visualized in *R* using the package *ggplot2*^57^.

## Results

### Whale recaptures across and between oceans

Combining fluke imagery and sighting data from Brazil (Breeding Stock A) and South Africa (Breeding Stocks B2 and C1), a total of 6982 individual humpback whales were photo-identified during 9730 sightings recorded from 1989 to 2022. In Brazil, 5616 individual whales were identified in 7998 sightings from 1989 to 2022. In South Africa, 1366 individual whales were identified in 1732 sightings documented from 2002 to 2022.

**Figure 1:**
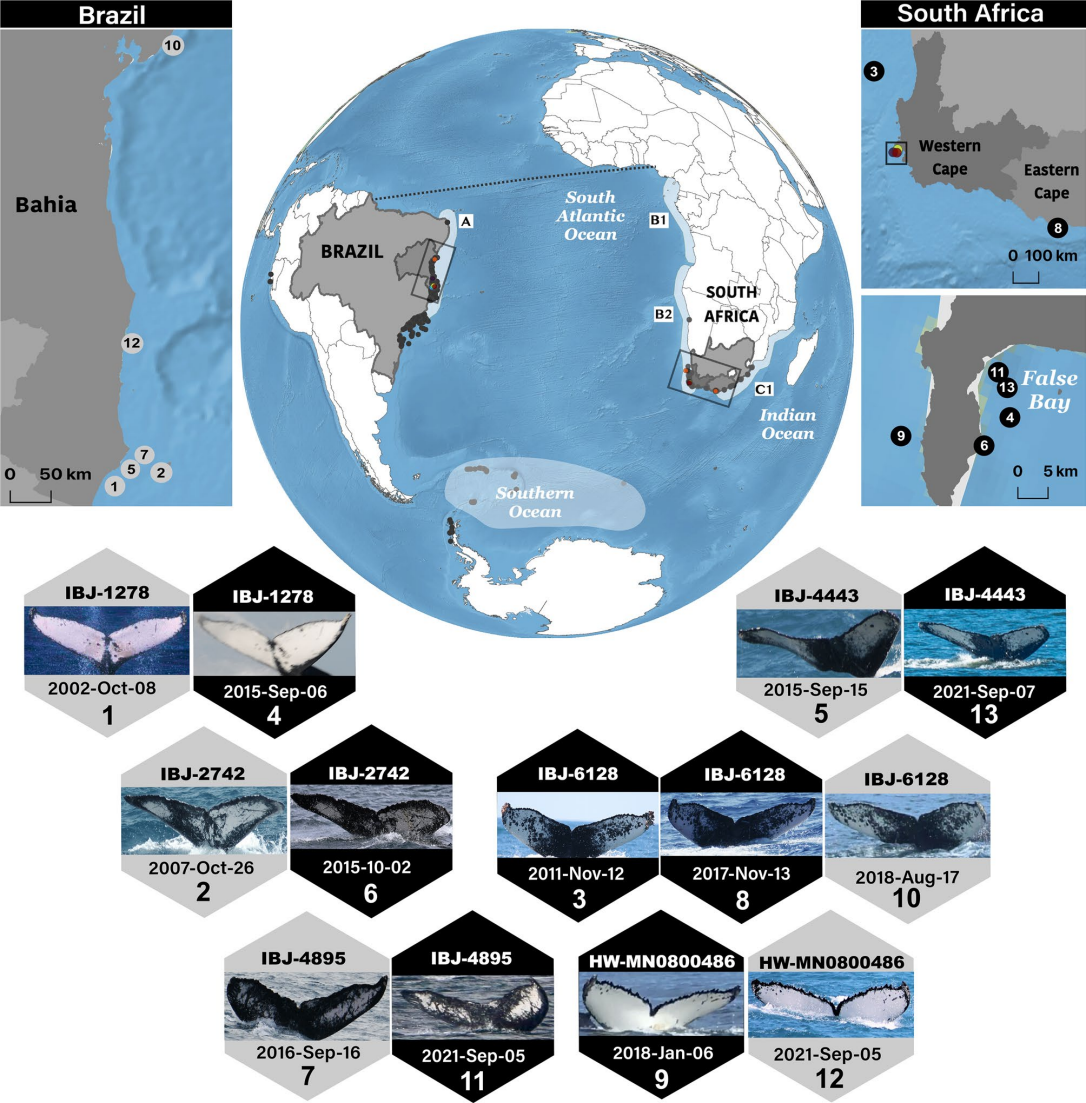
Maps of sighting locations and fluke images of six Southern Hemisphere humpback whales (*Megaptera novaeangliae*) photographed across the South Atlantic Ocean and Southwestern Indian Ocean (with the indication of the respective Breeding Stock or substock, A, B1, B2, and C1). The top left inset map shows whale sightings along the Atlantic coast of Brazil and the top right inset maps depicts sightings in the Western and Eastern Capes of South Africa (top) and zoomed in view of sightings in and outside of False Bay. Fluke images of each identified whale in the hexagons are numbered with sightings in Brazil colored gray and in South Africa colored black. The white oval in the Southern Ocean shows the Atlantic sector (Area III) of the Antarctica feeding grounds visited by humpback whales from Africa. Maps were created in QGIS version 3.28.3 (www.qgis.org).

Six adult humpback whales were individually photographed between Brazil and South Africa from 2002 to 2021 (Table 1; Fig. 1). Four humpback whales (IBJ-1278, IBJ-4443, IBJ-4895, IBJ-2742) first photographed off the Atlantic coast of Brazil from 2002 to 2016 were later photographed off the Western Cape province of South Africa from 2015 to 2021 (Table 1). Two whales (HW-MN0800486, IBJ-6128) first photographed in South Africa between 2011 and 2018 were photographed in Brazil from 2018 to 2022. The sighting of 6 whales represented (0.09%) of all identified individuals in both areas: 0.10% and 0.04% of all identified individuals in Brazil and South Africa respectively.

**Table 1:** Information on sightings of 6 Southern Hemisphere humpback whales photographically recaptured off the Abrolhos Bank of Bahia, Brazil and along the Western and Eastern Capes of South Africa. Time between captures is displayed as number of days (decimal years).

ID	Date	Sight. no	Country	Ocean	Time between recaptures [days (decimal years)]	Breeding stocks involved
HW-MN0800486	2018-Jan-06	9	South Africa	South Atlantic	–	A/B
HW-MN0800486	2021-Sep-05	12	Brazil	South Atlantic	1338 (3.67)	A/B2
IBJ-1278	2002-Oct-08	1	Brazil	South Atlantic	–	A/B2
IBJ-1278	2015-Sep-06	4	South Africa	South Atlantic	4716 (12.92)	A/B2
IBJ-2742	2007-Oct-26	2	Brazil	South Atlantic	–	A
IBJ-2742	2015-Oct-02	6	South Africa	South Atlantic	2899 (7.90)	B2
IBJ-4443	2015-Sep-15	5	Brazil	South Atlantic	-	A/B2
IBJ-4443	2021-Sep-07	13	South Africa	South Atlantic	2184 (5.98)	A/B2
IBJ-4895	2016-Sep-16	7	Brazil	South Atlantic	–	A/B2
IBJ-4895	2021-Sep-05	11	South Africa	South Atlantic	1815 (4.97)	A/B2
IBJ-6128	2011-Nov-12	3	South Africa	South Atlantic	–	A/B2/C1
IBJ-6128	2017-Nov-13	8	South Africa	Southwestern Indian	2193 (6.01)	A/B2/C1
IBJ-6128	2018-Aug-17	10	Brazil	South Atlantic	277 (0.76)	A/B2/C1

Five of the whales were photographed twice (once in Brazil and once in South Africa) and one whale was photographed three times (once in Brazil and twice in South Africa, on the east and west coast). In Brazil, matched individuals were photographed during the Southern Hemisphere breeding season (i.e., from June to November^39,58^.

### Trends in identification in Brazil and South Africa

**Figure 2:**
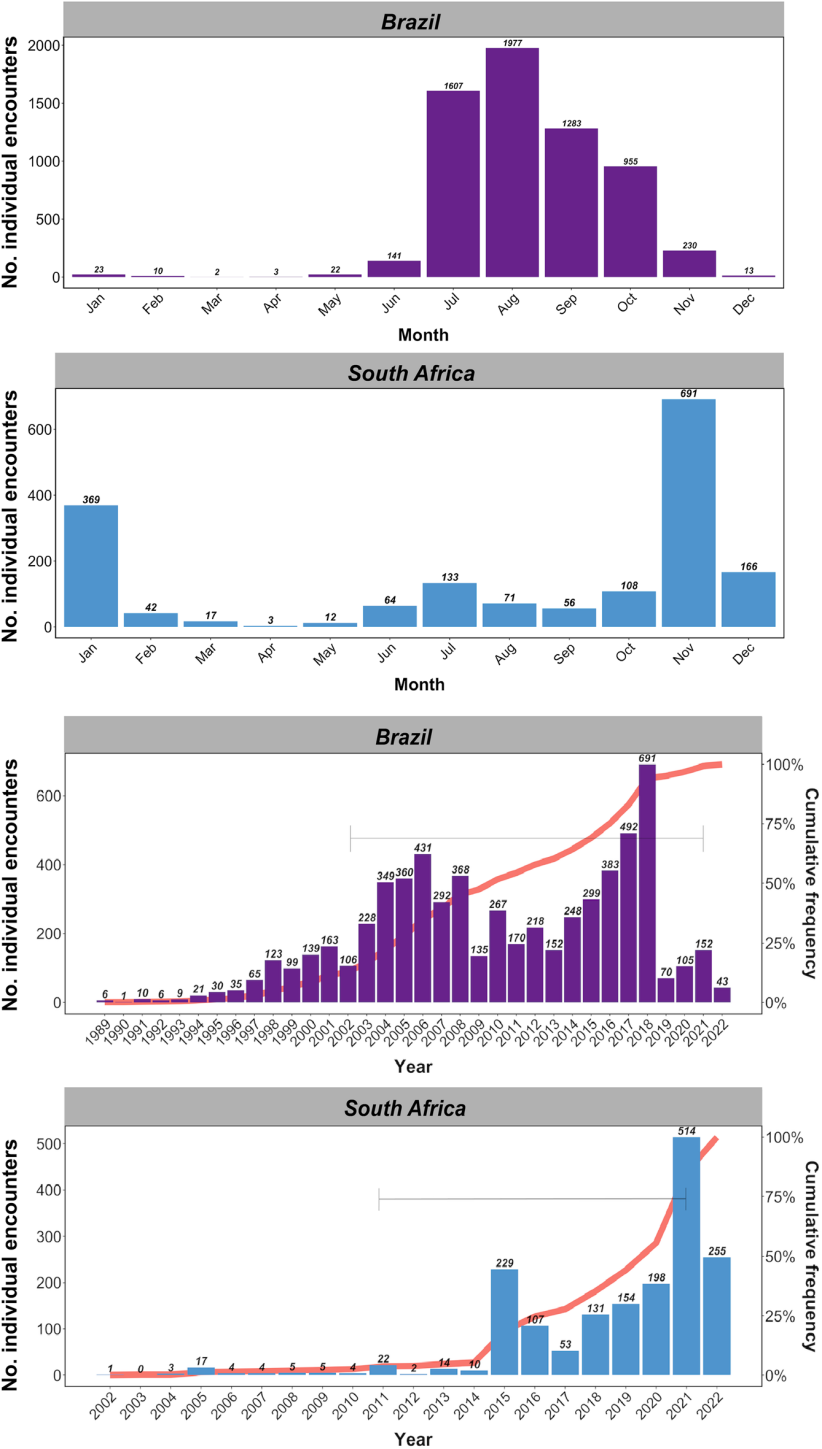
Bar charts showing the number of individual encounters of Southern Hemisphere humpback whales photographed by month and by year off the Atlantic coast of Brazil from 1989 to 2022 and off the Western and Eastern Capes of South Africa from 2002 to 2022. The horizontal gray bars in the yearly bar charts show the timespan between photo-ID recaptures of humpback whales in each region. The red line shows the cumulative frequency or ‘discovery curve’ of identified individuals. The graph was made in *R* statistical software^56^ using the package *ggplot2*^57^.

The number of identified whales increased dramatically in Brazil from 2002 to 2018 (Fig. 2) and the cumulative frequency of identified individuals reached an asymptote from 2017 to present (i.e., most of the population has been photo-identified) (Fig. 2). The increase in identified animals over time was more dramatic in South Africa: 0–23 whales were photographed per year from 2002 to 2015, and 53 to 514 whales identified per year from 2015 to 2022 (Fig. 2). Individual whales were identified between one and eight times in South Africa and between one and seven times in Brazil, with most individuals photographed once. A small percentage of whales were photographed more than 3 times in both locations.

## Discussion

The interchange of Southern Hemisphere humpback whales amongst Breeding Stocks highlights migratory plasticity in their use of the grounds of multiple stocks. The currently recognized boundaries of the different humpback whale stocks were developed through the assessment of catch histories from the 1930’s to the 1960’s with the Discovery Mark Programme in combination with other knowledge of catch and migration timing and more recently photographic mark-recapture and genetics^5,6,31,38,59,60^. These stock boundaries are further supported by the results of population genetics with biopsy samples from thousands of whales globally^10,12,61^. However, there are a growing number of accounts of whales undertaking long-distance migrations between the breeding grounds and feeding grounds of different stocks^11,21,25,28^.

The photographic recapture of several Southern Hemisphere humpback whales in the grounds for three breeding stocks over a 20-year period supports previous findings illustrating low levels gene flow and interchange of whales across the South Atlantic and into the Southwest Indian Ocean. For example, a large-scale assessment of population structure in Southern Hemisphere humpback whales (based on genetic sampling of 1527 individuals) demonstrated while gene flow was low between populations across the Southeastern Atlantic Ocean relative to gene flow of Southwestern Indian Ocean humpbacks, the exchange and mixing of individuals does occur between Breeding Stocks A, B, and C, with the lowest rates between B and C^10^.

Analysis of historical whaling records and long-term satellite tracking of humpback whales off the Abrolhos Bank revealed whales maintained fidelity to specific migratory routes to Antarctic feeding sites and used a limited number of routes despite shifting oceanographic conditions^32^. It is likely that these ‘extralimital’ movements are occurring at a low enough level to be unnoticed and occur at low enough rates (e.g., 1–2 per annum:^10^) as to not significantly influence genetic relationships between these populations^10^. Similarly, the growth of Southern Ocean humpback whale populations may result in increased overlap and migratory exchange between stocks and photographic recaptures^33,36^.

The small proportion of identified whales in each population recaptured across the South Atlantic and Southwestern Indian Oceans suggests most whales of these populations maintain fidelity to their original stock boundaries^10^. The limited number of resightings (2–3) of each humpback whale recaptured in our analysis prevented us from investigating the life histories of these individuals and determining if the observed movements represented a one-way shift in breeding grounds, or instead, the regular use of multiple breeding grounds over the life of individuals. The smaller relative size of the uploaded catalog of humpback whales on Happywhale from South Africa compared to the catalog from Brazil creates a clear bias in our ability to interpret the likely proportion of whales in these populations exhibiting these kinds of movements. Additionally, increased research efforts in both areas drove dramatic increases in whale sightings over time which could bias the interpretation of population sighting rates and likely increased the probability of our recaptures (see Fig. 2). Our findings on population trends should be considered preliminary as they are based on a growing database that includes datasets with varying levels of survey effort. Therefore, caution should be exercised when interpreting these results. Future efforts processing fluke imagery from different regions will facilitate assessments of the overlap between other Breeding Stocks in the South Atlantic Ocean, for example, between the adjacent substocks B1 and B2 and between substocks C1, C2, and C3.

Our findings highlight that despite high-fidelity of most population members to their original breeding stocks, individual whales can display patterns that deviate from the pattern in the stock. These deviations could bias estimates of population fidelity to areas and potentially could signal higher rates of shifting between breeding and feeding grounds that previously thought. For example, the use of multiple breeding grounds by IBJ-6128 suggests it exhibits more flexible patterns of migratory movements than other whales in these stocks, sharing breeding grounds of different stocks without needing to make large-distance migratory movements. In addition, its sighting in the feeding grounds of whales from substock B2 in the Benguela Current Large Marine Ecosystem off western South Africa indicates some whales may capitalize on nearby opportunities for feeding as substocks B and C are believed to join in the documented feeding aggregations in the area^62^.

Ongoing studies are needed to understand if large-scale changes in oceanic conditions in the Southern Hemisphere associated with climate change and/or population expansion drive humpback whales to adapt to temporal and spatial variation in environmental conditions by using multiple different breeding or feeding habitats in different years. For example, two humpback whales from Breeding Stock G first photographed in Ecuador were found in the breeding grounds of stock A off the Abrolhos Bank, Brazil during El Nino years^25^. Similarly, humpback whales normally persistently present in the ASSO, were (acoustically) absent from the region during 5 El Nino years suggesting whales shifted migration patterns based on sea and prey conditions^63^. Also, six humpback whales from stock A were found in Western Antarctic Peninsula, in the feeding area of stock G^11^. Evidence is still too limited to know whether it was changes in oceanographic conditions, population growth or advances in photo-ID analysis techniques that made it possible to identify changes in the migratory movements of humpback whales.

The migratory movements of humpback whales we report should be considered in estimates of abundance, stock boundaries, and local and regional management measures. Despite being based on a small number of whales, identification of these kinds of exchanges reinforce the importance of international cooperation to the conservancy of whales. This connection across the South Atlantic supports proposals of a South Atlantic Whale Sanctuary (SAWS) by Brazil and South Africa to the IWC^64^ and should stimulate more collaborative efforts of countries that share the South Atlantic Ocean. It also informs the Convention of Migratory Species (CMS) on humpback whale exchanges across stocks previously unidentified in the scientific literature, as for example, in seabirds^65^. Future work integrating regional photo-ID and genetic information on humpback whale populations with existing global datasets will improve our ability to detect these kinds of exchanges and to determine if their frequency is increasing or an artefact of increased research efforts and the development of tools to detect exchanges.

## Conclusions

Studying large-scale movements of megafauna populations crossing entire ocean basins is logistically very challenging and requires a combination of multiple methods, long-term studies, and a high degree of collaboration. Our findings provide additional support for the strength of collaborations between groups responsible for long-term photo-ID efforts in different regions to identify connections between humpback whale populations previously believed to be distinct. To our knowledge, this is the first comprehensive analysis of individual humpback whale movements across multiple locations in the South Atlantic Ocean, based on photographic mark-recapture of whale flukes. The availability of automated identification of individuals from thousands of whale fluke images has significantly improved the ability to rapidly identify whales and match those to global data sets within seconds. This ease of use and the open access nature of Happywhale has significantly increased the pool of potential data providers to these studies, extending photo-ID from a niche tool exclusive to scientific researchers, to allow for easy inclusion of opportunistic data from commercial whale-watching companies and citizen scientists^66^. The range of tools and information currently available is providing substantial insights into the population structure and degree of movements between recovering populations of whales and can give light into potential changes in their distribution and migration over time.

## Data Availability

The datasets generated and analyzed during this study are available from the corresponding author on reasonable request.
